# Modelling the effect of SMP production and external carbon addition on S-driven autotrophic denitrification

**DOI:** 10.1038/s41598-022-10944-z

**Published:** 2022-04-29

**Authors:** Grazia Guerriero, Maria Rosaria Mattei, Stefano Papirio, Giovanni Esposito, Luigi Frunzo

**Affiliations:** 1 Department of Mathematics and Applications “R. Caccioppoli”, Via Cintia, Monte S. Angelo, 80126 Naples, Italy; 2 Department of Civil, Architectural and Environmental Engineering, University of Naples Federico II, Via Claudio 21, 80125 Naples, Italy

**Keywords:** Environmental biotechnology, Environmental sciences

## Abstract

The aim of this study was to develop a mathematical model to assess the effect of soluble microbial products production and external carbon source addition on the performance of a sulfur-driven autotrophic denitrification (SdAD) process. During SdAD, the growth of autotrophic biomass (AUT) was accompanied by the proliferation of heterotrophic biomass mainly consisting of heterotrophic denitrifiers (HD) and sulfate-reducing bacteria (SRB), which are able to grow on both the SMP derived from the microbial activities and on an external carbon source. The process was supposed to occur in a sequencing batch reactor to investigate the effects of the COD injection on both heterotrophic species and to enhance the production and consumption of SMP. The mathematical model was built on mass balance considerations and consists of a system of nonlinear impulsive differential equations, which have been solved numerically. Different simulation scenarios have been investigated by varying the main operational parameters: cycle duration, day of COD injection and quantity of COD injected. For cycle durations of more than 15 days and a COD injection after the half-cycle duration, SdAD represents the prevailing process and the SRB represent the main heterotrophic family. For shorter cycle duration and COD injections earlier than the middle of the cycle, the same performance can be achieved increasing the quantity of COD added, which results in an increased activity of HD. In all the performed simulation even in the case of COD addition, AUT remain the prevailing microbial family in the reactor.

## Introduction

During the last few years, nitrate removal through sulfur-driven autotrophic denitrification (SdAD) has been thoroughly investigated. Compared to conventional heterotrophic denitrification, SdAD allows lower sludge production and reduced N_2_O emissions^1^, but results in the production of high sulfate concentrations as main shortcoming^2^. Among the various forms of S-based electron donors already studied, elemental sulfur is of economic interest despite the need to solubilize it prior to being effectively taken up by the microorganisms^1,3^.

The growth of autotrophic microbial families using elemental sulfur as an electron donor often comes with the natural growth of heterotrophic families. The latter might be already present in the influent wastewater or proliferate due to the natural production of organic matter associated with the autotrophic microbial activities^4,5^. Notwithstanding, literature lacks SdAD studies focused on the production and consumption of these autotrophically-generated organic compounds from a kinetic point of view. As reported by Laspidou and Rittmann^6^ and Ni et al.^7^, the organic matter is mainly composed by soluble microbial products (SMP) which are the result of autotrophic and heterotrophic microbial activities^8^. The SMP produced during SdAD are made up of organic byproducts associated with biomass metabolism and decay^7,9,10^. In particular, part of SMP comprises a readily available carbon source, i.e. biomass associated products (BAP) and utilization-associated products (UAP), while another part is made up of extracellular polymeric substances (EPS) and stocked biodegradable substances (STOB), which need a preliminary hydrolysis step to become bioavailable^11^. During the SdAD maintained by mixed microbial consortia, the most relevant heterotrophic microorganisms are heterotrophic denitrifying (HD) and sulfate reducing bacteria (SRB)^4,5,12–14^. The presence of HD was evident in full scale SdAD studies in the presence of chemical oxygen demand (COD)^15^ and in experimental studies conducted under feast-famine conditions^16^. Also, Qiu et al.^17^ and Oh et al.^18^ highlighted the possibility to add various organic compounds to a denitrifying autotrophic microbial consortium aiming to improve the process efficiency and to reduce the sulfate output without modifying the optimal working parameters. The favorable effects of organic supplementation on the performance of SdAD has not been attributed to the role of SRB but rather to the activity of HD, which considerably reduce the nitrate loading on autotrophic denitrifiers^18,19^. SMP represent an alternative source of organic matter which could contribute to the heterotrophic-associated process refinement of SdAD with regards to nitrate removal and sulfate reduction by minimizing the external carbon addition.

In this framework, mathematical modelling is a useful tool to elucidate the competition among different microbial families and the performances in terms of nitrate removal and sulfate production occurring during the SdAD process. Indeed, previous mathematical models with elemental sulfur as electron donor have been mainly focused on evaluating the kinetics of the process in terms of nitrate removal^20–22^, nitrite accumulation^5^ and N_2_O emission^23,24^. In addition, the co-occurrence of SdAD and heterotrophic denitrification^17,25^ has been also modelled to investigate the same parameters. Other models have been carried out taking into account the concomitant removal of sulfide, carbon and nitrogen compounds. Sulfide has been used as sulfurous electron donor for autotrophic denitrifiers in those models and SMP production was not considered^26–29^. Moreover, none of the existing models has provided a thorough picture on the competition among different bacterial families (especially SRB) and the growth of these on SMP^30^.

In this context, this work proposes a mathematical model to study the concomitance of SdAD, as the main process, heterotrophic denitrification and sulfate reduction simply on UAP and BAP or in presence of an external carbon source. The processes were simulated in a sequencing batch reactor (SBR), which was chosen as bioreactor configuration to enhance the formation and the uptake of SMP by the heterotrophic families. Indeed, a SBR allows to operate under feast-famine conditions that are needed to let the active bacteria use the SMP as main organic source while promoting the accumulation of biomass^11^. When an external source of organics was considered, the timing of supplementation of such carbon source was investigated, being this of crucial importance due to the presence of two heterotrophic families. This is another aspect that makes the model here proposed novel compared to the existing ones. The main objectives of the presented can be summarized as follows:

- To assess the influence of the addition of an external carbon source on nitrate removal via HD and sulfate reduction via SRB, also to refine the concentration in the effluent;
- To reduce the concentration of sulfate in the effluent related to the SdAD, which represent the main disadvantage of the process;
- To analyze the competition between the different microbial families involved in the model;
- To evaluate the SMP production and consumption and analyze whether these compounds can support part of the heterotrophic-associated processes which occur during SdAD.

## Biological model

**Figure 1:**
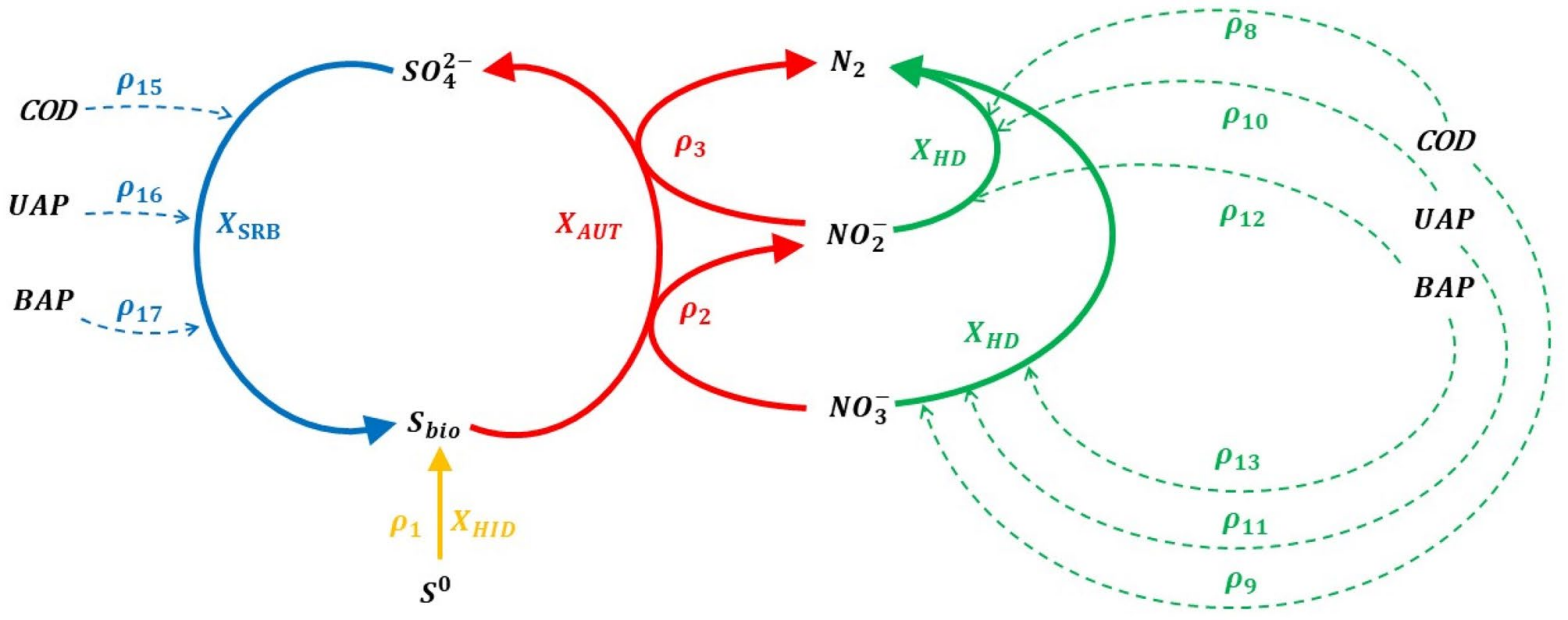
Schematic representation of the biological processes considered in the proposed model: sulfate reduction maintained on external COD, BAP and UAP (blue), sulfur-based autotrophic denitrification (red), heterotrophic denitrification maintained on external COD, BAP and UAP (green), elemental sulfur hydrolysis (yellow). Each ${\uprho }_{{\text{i}}}$ represent a term of ith-reaction.

The three main processes considered in the mathematical model are SdAD with elemental sulfur, heterotrophic denitrification and sulfate reduction. As shown in Fig. 1, HD and SRB were assumed to grow on UAP, BAP and external COD (Fig. 1).

Elemental sulfur (S^0^) is assumed to be supplemented in the form of “lentils” so it requires a preliminary hydrolysis step for its conversion into a more bioavailable form (S_bio_). This transformation is microbially-catalyzed by hydrolytic biomass (HYD), which was considered for the first time by Kostrytsia et al.^5^ and indicated in yellow in Fig. 1 ($\rho_{1}$). S^0^ lentils are supposed to constantly remain in the reactor after each SBR cycle (see section “Numerical simulations”), representing a support for the growth of the different microbial families involved and a continuous source of S_bio_ over time^11^. The S_bio_ generated by S^0^ hydrolysis was assumed to remain within the solid S^0^ lentils^4,31,32^ and, thus, not escaping the reactor contrary to the soluble substrates (i.e. NO_3_^−^, NO_2_^−^, SO_4_^2−^).

When S_bio_ is consumed, SdAD is assumed to undergo a two-step process consisting of a first transformation from NO_3_^−^ to NO_2_^−^ ($\rho_{2}$,) and a second from NO_2_^−^ to N_2_ ($\rho_{3}$). The intermediate steps allowing the production of NO and N_2_O were not considered, assuming the occurrence of optimal conditions in the system that allow those steps not to be limiting^33^. Sulfate reduction is carried out by SRB, which transform the sulfate produced by the denitrifying autotrophs into sulfide (S^2−^), using all the carbon sources available (i.e. UAP, BAP and external COD). The sulfide produced is assumed to be used again by autotrophic denitrifying bacteria as S_bio_. This assumption is possible being the typical sulfide-driven autotrophic denitrification kinetics faster than those obtained with S_bio_^1^, and not limiting for the SdAD process here considered. Heterotrophic denitrification is assumed to occur on all bioavailable organic compounds (BAP, UAP, COD) and convert NO_3_^−^ ($\rho_{9} , \rho_{11} , \rho_{13}$) and NO_2_^−^ ($\rho_{8}$,$\rho_{10}$,$\rho_{12}$) into N_2_.

**Figure 2:**
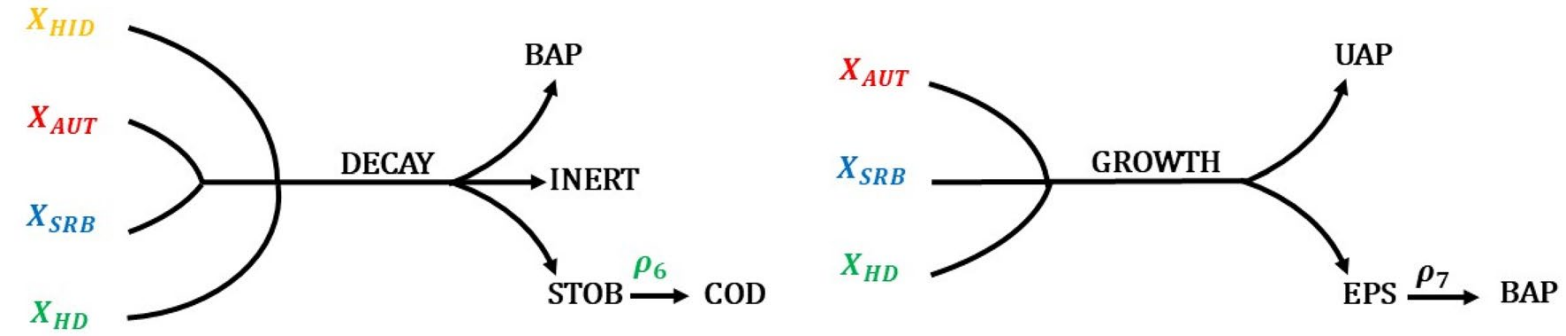
Endogenous processes of microbial families and production of SMP deriving from microbial activities. The biomass decay (left-hand side) associated with the hydrolytic (${\text{X}}_{{{\text{HID}}}}$), autotrophic denitrifying (${\text{X}}_{{{\text{AUT}}}}$), heterotrophic denitrifying (${\text{X}}_{{{\text{HD}}}}$) and sulfate reducing (${\text{X}}_{{{\text{SRB}}}}$) families releases BAP, INERT and STOB, with the latter being hydrolyzed into bioavailable COD by reaction ${\uprho }_{6}$. During the growth of the microbial families (right-hand side), denitrifying autotrophs (${\text{X}}_{{{\text{AUT}}}}$), denitrifying heterotrophs (${\text{X}}_{{{\text{HD}}}}$) and SRB (${\text{X}}_{{{\text{SRB}}}}$) produce UAP and BAP, with the latter deriving from the hydrolysis of EPS as regulated by reaction ${\uprho }_{7}$.

To take into account the natural production of organics from the growth and decay of biomass is considered that all the microbial families involved lead to the production of SMP (Fig. 2). In particular, BAP, inert material and STOB are released during the decay of the microbial families. With regard to the microbial growth, denitrifying autotrophs and heterotrophs as well as SRB result in the production of UAP and EPS, which are further solubilized into BAP prior to being bioavailable for the heterotrophs. The hydrolytic biomass was excluded from the production of SMP during the growth phase due to a lack of information regarding the process in which it is involved.

## Mathematical model

A differential model describing the dynamics of autotrophic, denitrifying heterotrophs and sulfate reducing bacteria is formulated based on mass balance considerations. The model considers seven different biomasses ${\text{X}}_{{\text{i}}} ,{\text{ i}} = \left[ {{\text{HYD}},{\text{ AUT}},{\text{ STO}},{\text{ EPS}},{\text{ HD}},{\text{ SRB}},{\text{ I}}} \right]{ }$ and nine different compounds ${\text{S}}_{{\text{j}}} ,{\text{j}} = [{\text{S}}_{0}$, ${\text{S}}_{{\text{bio}}}$, ${\text{NO}}_{3}^{ - } ,{\text{ NO}}_{2}^{ - }$, ${\text{N}}_{2} ,$ ${\text{SO}}_{4}^{2 - }$, ${\text{UAP}}$, ${\text{BAP}}$, ${\text{COD}}]$. The four active biomasses are represented by: ${\text{X}}_{{{\text{HYD}}}} { }$ which is the hydrolyzing biomass responsible of the transformation of elemental sulfur into bioavailable sulfur, ${\text{X}}_{{{\text{AUT}}}}$ autotrophic denitrifying biomass that uses sulfur as electron donor, ${\text{X}}_{{{\text{HD}}}}$ heterotrophic biomass that uses different types of organic matter as substrate, ${\text{X}}_{{{\text{SRB}}}}$ sulfate reducing bacteria which also use all the organic matter as substrate to reduce sulfate. The inactive biomasses are: ${\text{X}}_{{{\text{EPS}}}}$, ${\text{X}}_{{{\text{STO}}}}$ and inert material ${\text{X}}_{{\text{I}}}$ derived from the biomass decay. The substrates involved in the model are: ${\text{ S}}_{{{\text{S}}^{0} }}$, ${\text{ S}}_{{{\text{S}}_{{\text{bio}}} }}$, ${\text{S}}_{{{\text{NO}}_{3}^{ - } }}$, ${\text{ S}}_{{{\text{NO}}_{2}^{ - } }}$ , ${\text{S}}_{{{\text{N}}_{2} }} ,{\text{ S}}_{{{\text{SO}}_{4}^{2 - } }} ,{\text{ S}}_{{{\text{UAP}}}}$, ${\text{ S}}_{{{\text{BAP}}}}$, ${\text{S}}_{{{\text{COD}}}}$. The biomasses ${\text{X}}_{{\text{i}}}$ and substrates ${\text{S}}_{{\text{j}}}$ interact according to the biological processes described in the previous Section.

The mathematical model developed in the present work is made up by a system of first order impulsive ordinary differential equations (IDEs), which are used to model the biological processes described in the previous section occurring in a SBR configuration. Indeed, such equations are well-suited to model processes that are continuous under most conditions but undergo instantaneous changes. An impulsive differential equation is described by three components: a continuous-time differential equation, which governs the state of the system between impulses; an impulse equation, which models an impulsive jump defined by a jump function at the instant an impulse occurs; and a jump criterion, which defines a set of jump events in which the impulse equation is active^34^. The main features of the SBR configuration which are repeated for each cycle after the first initial filling have been considered as follows:

1. First Reaction period (continuous)
2. Injection of COD (instantaneous)
3. Secondo Reaction period (continuous)
4. Settling (instantaneous)
5. Emptying (instantaneous)
6. Filling (instantaneous)

In the present study, the settling, emptying and refilling processes were approximated by an instantaneous change of state of the system, which occurred at a prescribed time dictated by the duration of the combined cyclical reaction phases. The duration of each cycle is denoted as $\tau$ and the time of injection of COD is denoted as $\tau_{d} \le \tau$. The model is described by the following impulsive differential equations for both substrates and microbial species:

1 $$\dot{S}_{j} \left( t \right) = r_{S,j} \left( {t,{\varvec{S}},{\varvec{X}}} \right), \,\,t \in J = \left[ {0,T} \right] , \,\,\,t \ne t_{k} ,\,\,S_{j} \left( 0 \right) = S_{j0} ,$$

2 $$\dot{X}_{i} \left( t \right) = r_{X,i} \left( {t,{\varvec{S}},{\varvec{X}}} \right),\,\, t \in J = \left[ {0,T} \right] , \,\,\, t \ne t_{k} ,\, X_{i} \left( 0 \right) =X_{i0} ,$$

3 $$\Delta S_{j} \left( {t_{k} } \right) = - \alpha_{j} S_{j} \left( {t_{k}^{ - } } \right) + \alpha_{j} S_{in,j} ,\,\,\,\,k = 1, \ldots .,m,$$

4 $$\Delta X_{i} \left( {t_{k} } \right) = - \gamma_{i} X_{i} \left( {t_{k}^{ - } } \right) ,\,\,\,\,\,k = 1, \ldots .,m,$$

where $S_{j} \left( t \right), { }X_{i} \left( t \right)$ are the jth substrate and the ith biomass concentrations at time t respectively; $r_{S,j}$ and $r_{X,i}$ are the reaction terms for the jth substrate and the ith biomass. $S_{j0}$ and $X_{i0}$ are the initial concentration within the reactor for the jth substrate and the ith biomass; $S_{in,j}$ is the concentration of the jth substrate in the fresh influent; 0 = $t_{0} < t_{1} < \cdots < t_{m} < t_{m + 1} = T,$ $t_{k + 1} - t_{k} = \tau ,$ where $\tau$ denotes the duration of each cycle, $\Delta S_{j} \left( {t_{k} } \right) = S_{j} \left( {t_{k}^{ + } } \right) - S_{j} \left( {t_{k}^{ - } } \right)$, $\Delta X_{i} \left( {t_{k} } \right) = X_{i} \left( {t_{k}^{ + } } \right) + X_{i} \left( {t_{k}^{ - } } \right)$ , with $S_{j} \left( {t_{k}^{ + } } \right)$, $X_{i} \left( {t_{k}^{ + } } \right)$, $S_{j} \left( {t_{k}^{ - } } \right)$, $X_{i} \left( {t_{k}^{ - } } \right)$ , being the right and left limits of $S_{j} \left( t \right)$ and $X_{i} \left( t \right)$ at time $t = t_{k} ;$ $\alpha_{j}$ represents the emptying/refilling ratio and $\gamma_{i}$ takes into account the fraction of biomass removed from the system during the emptying phase.

The $r_{S,j}$ and $r_{X,i}$ are expressed as a combination of the kinetic terms and stoichiometric parameters reported in the Tables S.1, S.2 and S.4 of the supplementary information.

For all the biomasses the coefficient $\gamma_{i}$ is considered equal to zero simulating a settling process with 100% efficiency. The dilution is only applied to the substrates which are considered in dissolved form:$\user2{ }S_{{SO_{4}^{2 - } }}$,$S_{UAP}$**,** ${ }S_{BAP}$, $S_{{{\text{COD}}}}$***,*** $S_{{NO_{3}^{ - } }}$, ${ }S_{{NO_{2}^{ - } }}$ . All the other compounds included in the model are supposed to undergo a complete sedimentation. According to Sierra-Alvarez et al.^24^ and Liu et al.^35,36^, in the present model is assumed that the bioavailable sulfur cannot be washed out during the emptying phase since it is supposed to be retained within the microbial sludge. The value of $\alpha_{j}$ is set equal for all the dissolved substrates.

The first and second reaction periods are discriminated by the time of soluble COD injection in the system. Such operation is considered to occur instantaneously and does not affect the concentration of the other compounds.

5 $$\left\{ {\begin{array}{*{20}l} {\left( {k - 1} \right) \cdot \tau < t \le \left( {k - 1} \right)\tau + \tau_{d} \quad k = 1,...,n,} \hfill & {FIRST \,REACTION \,PERIOD} \hfill \\ {\left( {k - 1} \right) \cdot \tau + \tau_{d} < t \le k \cdot \tau \quad k = 1,...,n,} \hfill & {SECOND\, REACTION \,PERIOD} \hfill \\ \end{array} } \right.$$

The equation that defines the jump function for COD between the two reaction periods is:

6 $$\Delta S_{{{\text{COD}}}} \left( {\left( {k - 1} \right)\tau + \tau_{d} } \right) = S_{in,COD} ,\,\,\,k = 1,...,n$$

where $\tau$ represents the duration of each cycle, $\tau_{d}$ represents the duration of the first reaction period until the COD injection occurs, $n$ is the number of cycles which varies with respect to different retention times, $S_{in,COD}$ is the concentration of COD that must be reached in the reactor at that time.

## Process rates

The reaction terms in Eqs. (1) and (2) are formulated as Monod kinetics and their expressions are reported in Table S.4 in the supplementary material. According to Kostrytsia et al.^5^, elemental sulfur is not directly oxidized, but an hydrolysis step is taken into account to model its conversion to bioavailable sulfur. This conversion is modelled through a nonlinear reaction term, which depends on the concentration of the hydrolyzing biomass, the amount of available elemental sulfur and its mass specific area. Autotrophic denitrification takes place in two sequential steps: a first step from nitrate to nitrite and then from nitrite to molecular nitrogen. As the same biomass carries out both denitrification steps, a fractional term is introduced. According to Huiliñir et al.^22^, the inhibition term regarding nitrite accumulation is not considered.

To take into account that the same biomass cannot work simultaneously on different substrates, fractional terms were added with respect to a classic Monod kinetics for the heterotrophic species growing on different carbon sources and nitrogen compounds. Due to the lack of experimental measurements of the specific EPS production from the different microbial family involved in the model, the same hydrolysis constant for the EPS produced was used^9^. Since no specific values are available regarding the use of BAP and UAP as organic substrates by SRB, the same reducing growth factor of HD on BAP and UAP are considered.

## Numerical simulations

The initial amount of sulfur is set to 21 g/L (Table 2) for each simulation. Consequently, for all the conditions investigated the final simulation time is set to 300 days, which is the evaluated time needed to achieve the complete solubilization of the initial elemental sulfur.

The simulations are carried out for three different scenarios depending on the duration of each SBR cycle and on the time of COD injection. Scenario I simulated the occurrence of the three main processes in the SBR in the absence of external COD and was used as reference. Scenario II was characterized by the injection of an amount of COD (380 mg/l) corresponding to the stoichiometric value needed to achieve a complete sulfate reduction^37^ and COD supplied in excess (i.e. 500 mg/l) to the stoichiometric value. For all scenarios, three different SBR cycle durations $\tau$ of 10, 15 and 20 days were investigated. The time of COD injection $\tau_{d}$ after the start of each cycle is varied depending on the duration of the cycle. The summary of all simulation parameters is shown in Tables 1 and 2.

**Table 1:** Resume of the simulations performed. The duration of each cycle and the time of COD injection are reported. The simulations in the presence of added COD were performed with both a stoichiometric COD amount and an excess of COD with respect to the complete sulfate reduction requirements.

Scenario	COD	Durations of the cycles $\tau$ (days)	Period after the injection of COD occurs
Scenario I	Absent	10-15-20	Not present
Scenario II	Stoichiometric and in excess with respect to the complete sulfate reduction	10-15-20	5-8-10-15

**Table 2:** Initial data for all the simulations performed.

$X_{HYD}$*(*mg/l)	$X_{AUT}$(mg/l)	$X_{STOB}$(mg/l)	$X_{EPS}$(mg/l)	$X_{HD}$(mg/l)	$X_{SRB}$(mg/l)	$X_{I}$(mg/l)		
500	1100	0	0	0.1	0.1	0		

$S_{{S_{0} }}$(mg/l)	$S_{{S_{bio} }}$(mg/l)	$S_{{NO_{3}^{ - } }}$(mg/l)	$S_{{NO_{2}^{ - } }}$(mg/l)	$S_{{N_{2} }}$(mg/l)	$S_{{SO_{4}^{2 - } }}$(mg/l)	$S_{UAP}$(mg/l)	$S_{BAP}$(mg/l)	$S_{COD}$(mg/l)
21,000	0	210	0	0	0	0	0	0

The initial data for each simulation are reported below:

The initial concentration of both ${\varvec{X}}_{{{\varvec{SRB}}}}$ and ${\varvec{X}}_{{{\varvec{HD}}}}$ is assumed to be considerably lower than that of autotrophic biomass, in order to better study their natural growth in an original autotrophic-dominated consortium.

## Results and discussion

### Scenario I: NO COD injection

**Figure 3:**
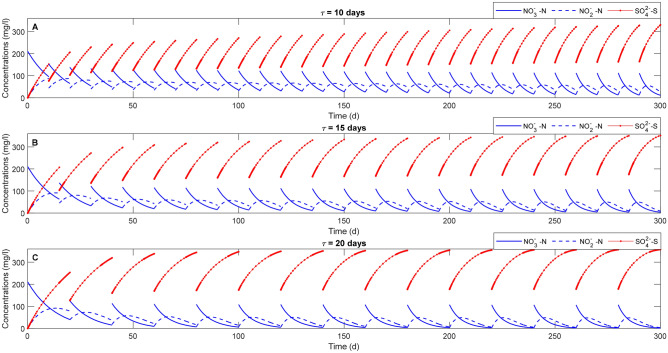
Autotrophic denitrification performances in terms of nitrate removal (solid blue), nitrite evolution (dashed blue) and sulfate production (solid red) with three different SBR cycle durations, ${\uptau }$ = 10 (**A**), 15 (**B**) and 20 (**C**) days, in the absence of external COD addition.

The numerical studies in Scenario I without any external COD supplementation are carried out to elucidate the effect of SMP production on the competition among the different microbial families and to serve as a reference to better highlight the effect of COD addition studied in the other two scenarios. Figure 3 shows the evolution of nitrate, nitrite and sulfate concentrations during SdAD for three different values of SBR cycle duration $\tau$.

It is possible to observe that an increase of $\tau$ leads to an increase of the nitrate removal efficiency, which results in a higher sulfate production. When $\tau$ is set at 15 days, the nitrate removal efficiency is close to 100% in the latest cycles (n > 14) and nitrite concentration approaches to zero after being accumulated up to approximately 50 mg/l. Conversely, the nitrate removal efficiency approaches to 100% only after 4 cycles when the duration of the cycle $\tau$ is 20 days. According to Kostrytsia et al.^5^, shorter retention times lead to a not complete removal of nitrogenous compounds. Indeed, higher NO_2_^−^ concentration is evident when the duration of the cycle is 10 days, when an incomplete denitrification process occurs.

**Figure 4:**
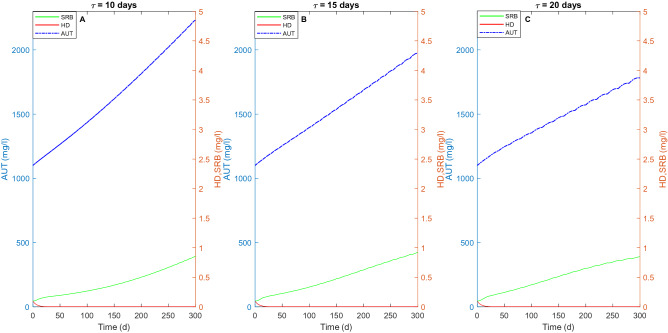
Evolution of the concentration (in mg COD/l) of autotrophs (blue line), heterotrophic denitrifiers (red line) and sulfate reducing bacteria (green line) overtime for three different durations of the SBR cycle (${\uptau }$ = 10, 15 and 20 days) in the absence of external COD.

The autotrophic biomass concentration increases in the reactor overtime for all the SBR cycle durations. In particular, the highest concentration of autotrophic biomass up to approximately 2200 mg COD/L is obtained when $\tau$ is 10 days (Fig. 4A). The higher biomass concentration at $\tau$ = 10 d is an indicator of the higher biomass activity, which is likely stimulated by the more frequent replacement of the influent solution. Moreover, comparing Figs. 3A and 4A it can be noticed that the increase of the concentration of autotrophic denitrifiers over time leads to a higher nitrogenous compounds removal. Although the higher biomass concentration, the process requires longer times to be completed. Regarding the two heterotrophic families, the results suggest that HD are less resistant than SRB for the whole cycle duration, with the latter being more capable to survive in the absence of external carbon source probably due to the presence of high sulfate concentrations (Fig. 4). In the absence of SRB (data not shown), HD are able to growth on the organic carbon deriving from the microbial activities, which is usually attributed to cell lysis or SMP as experimentally demonstrated by Wang et al.^4^.

**Figure 5:**
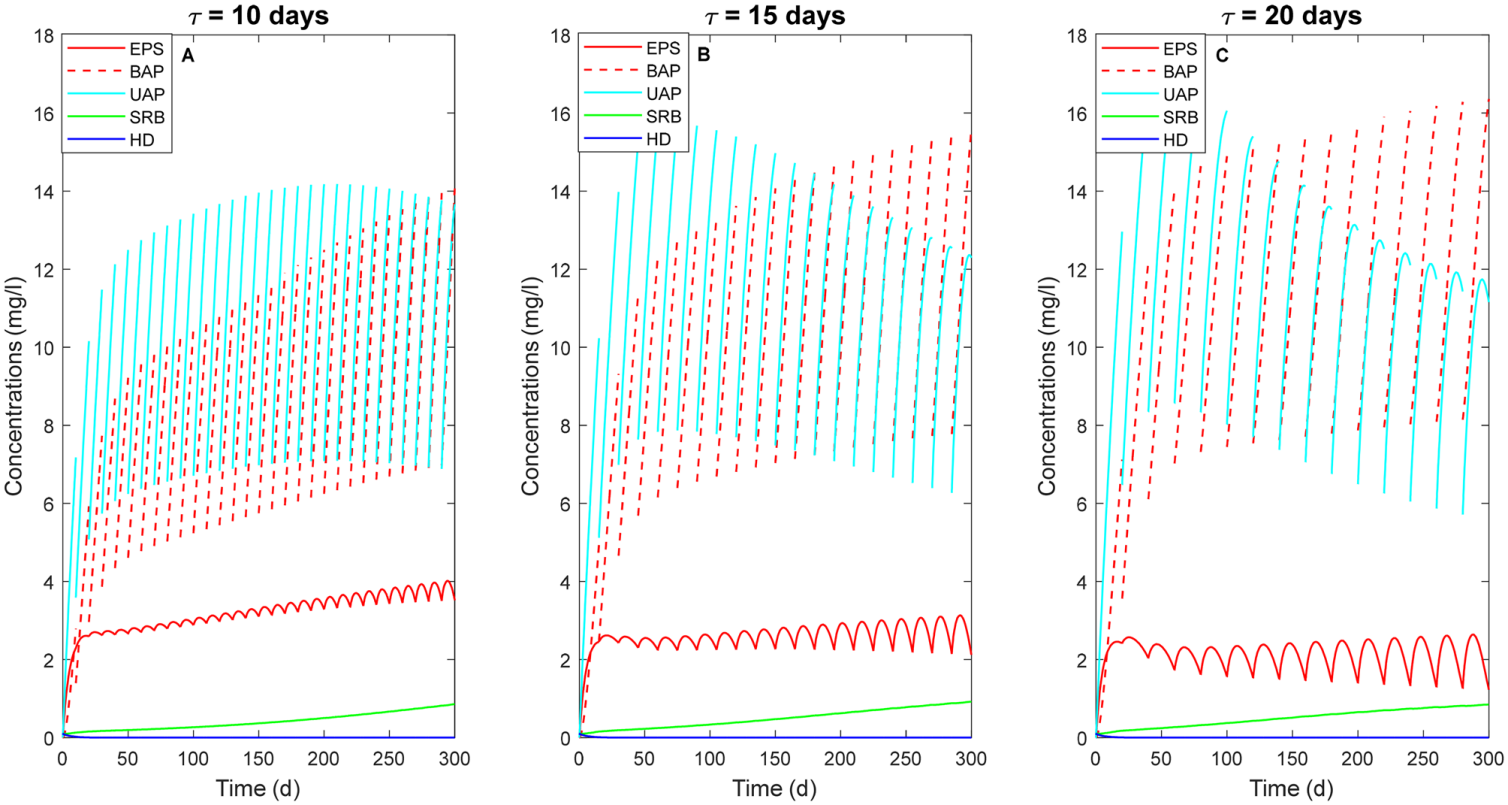
SMP production and concentration of heterotrophic families (i.e. HD and SRB) overtime with no external source of COD for three different values of ${\uptau }$ = 10 days (**A**), 15 days (**B**), 20 days (**C**). The solid dark blue line represents the heterotrophic denitrifiers (HD), while the solid green line indicates the sulfate-reducing bacteria (SRB). The solid red line indicates the EPS, which leads to the production of BAP (red dashed line) after hydrolysis. The solid light blue line represents the UAP.

The increase of the biomass activity over time reported in Fig. 4 results in an increased SMP production, as shown in Fig. 5. The highest EPS concentration is obtained for the shortest cycle duration and reaches approximately 3.65 mg/l (Fig. 5A) at the end of the simulation period. However, comparing the simulations carried out with the three different $\tau$ values, it can be noticed that the production of both BAP and UAP is higher at longer $\tau$. In particular, the increase of the BAP production over time is due to both EPS hydrolyzation and decay of the microbial families, which is higher at longer cycle durations. Furthermore, also the consumption of BAP and UAP is higher in the cases of longer cycles.

Figure 5 also shows that the denitrifying heterotrophic biomass is not able to grow on BAP and UAP and is outcompeted by both AUT and SRB, with the latter growing on the sulfate produced during SdAD. The SRB are likely able to grow on UAP and responsible for their degradation, confirming what was previously observed in experimental studies when SMP were used as electron donor for sulfate reduction under famine conditions^8,16^.

## Scenario II: COD Injection

### Evolution of nitrate, nitrite and sulfate at different COD amounts and injection times

**Figure 6:**
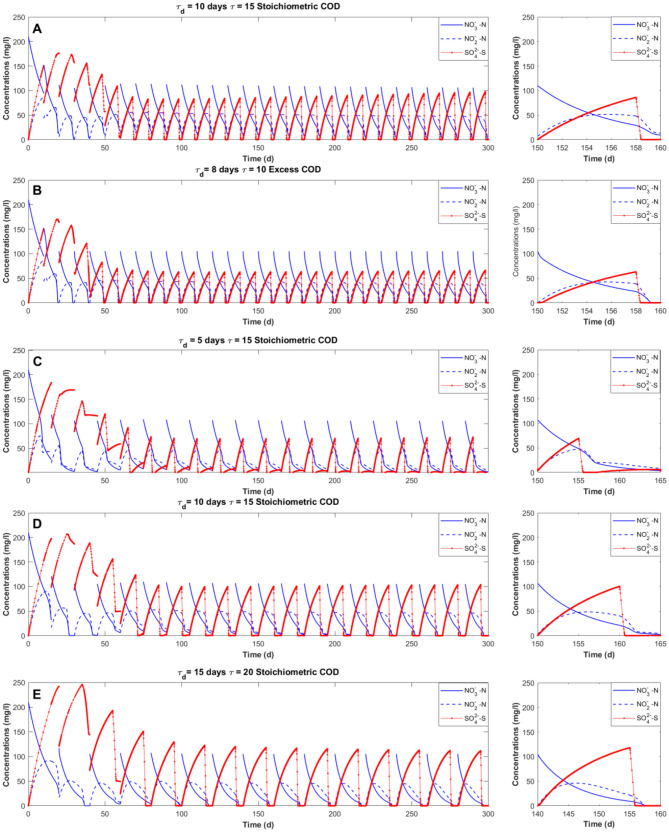
Nitrate removal (solid blue), nitrite evolution (dashed blue), sulfate production and consumption (solid red) in four different cases: (**A**) (${\uptau }$ = 10 days an injection of stoichiometric COD at ${\uptau }_{{\text{d}}}$ = 8 days), (**B**) (${\uptau }$ = 10 days an injection in excess of COD at ${\uptau }_{{\text{d}}}$ = 8 days), (**C**) (${\uptau }$ = 15 days an injection of stoichiometric COD at ${\uptau }_{{\text{d}}}$ = 5 days), (**D**) (${\uptau }$ = 15 days an injection of stoichiometric COD at ${\uptau }_{{\text{d}}}$ = 10 days), (**E**) (${\uptau }$ = 20 days an injection of stoichiometric COD at ${\uptau }_{{\text{d}}}$. = 15 days).

Figure 6 shows the effect of adding an external COD source on the three processes investigated at different $\tau$ and $\tau_{d}$ values. In each case, the system reaches a pseudo steady state after approximately 4–5 cycles and the effects of the COD injection, either in excess or stoichiometric, are positive for nitrate and sulfate removal.

Figure 6C,D highlight that the time of COD injection mainly affects the production and consumption of sulfate. Indeed, when COD is added prior to the complete nitrate removal by autotrophs, the growth of HD is favored due to their higher growth rate. From Fig. 6C it can be noticed that nitrate and nitrite (previously produced by SdAD) are quickly removed by heterotrophs when the addition of COD occurs on day 5, as shown by the change of the slope in nitrate evolution. A lower $\tau_{d}$ leads to a lower sulfate production since nitrate reduction is mainly performed by HD in the presence of external COD. A direct consequence of the consumption of the COD by the HD is the uncomplete sulfate reduction. It can be observed (Fig. 6C) that lower values of $\tau_{d}$ lead to a temporary increase of the sulfate concentrations when a stoichiometric amount of COD is added, since SRB are outcompeted by HD for the COD consumption. In both cases (Fig. 6C,D) the sulfate concentration attains very low values at the end of the SBR cycle after the attainment of the pseudo steady state. For longer $\tau$ (Fig. 6E), a higher percentage of nitrate is removed through autotrophic denitrification favoring sulfate production (6A and 6C).

For shorter SBR cycles ($\tau$ = 10 days), the addition of a higher amount of COD, with respect to the stoichiometric quantities (Fig. 6A), is required (Fig. 6B) to obtain a similar performance efficiency in terms of nitrate removal and sulfate reduction when longer cycle occurs. The excess of COD added is mainly used by HD, leading to a reduction of the time needed to obtain a complete denitrification and a lower sulfate production, as reported experimentally by Sahinkaya et al.^15^.

Furthermore, the addition of an external carbon source is observed to enhance heterotrophic denitrification resulting in a lower nitrite accumulation, which is reported to negatively affect SdAD^17,26,38^. The marginal presence of nitrite in the SBR here investigated justifies the choice of not considering any inhibition term on autotrophic denitrifiers due to nitrite in this model^39^.

It must be also reported that the simultaneous activity of AUT and HD was experimentally observed to decrease N_2_O accumulation and emission^25^, which is neglected in the present model. Previous studies also demonstrated that the addition of organic substances have a good influence on pH that makes the typical addition of limestone during SdAD unnecessary^18,40^.

With respect to the previous works, the time of COD injection is here investigated for the first time, as the addition of external carbon was previously considered only at the beginning of the process^25,41^. The variation of $\tau_{d}$ has a strong influence on the whole process (Fig. 6). For $\tau_{d} > \tau /2$, autotrophic denitrification prevails over heterotrophic denitrification resulting in lower treatment costs and sludge production. Conversely, for $\tau_{d} < \tau /2$, in particular for the shortest cycle duration ($\tau$ = 15 d), the removal efficiency of nitrate and sulfate decreases if a proper amount of COD is not injected. In addition, the choice of a proper time of COD addition can result in positive economic consequences as, for instance, a lower amount of COD can be supplemented to obtain optimal effluent nitrate, nitrite and sulfate concentrations. Furthermore, sulfate reduction enhanced by COD addition leads not only to the absence of sulfate in the effluent, but also to the possibility to reuse and consequently reduce the total sulfur used in the process. Indeed, based on the model assumption, the reduced sulfate is converted into sulfide that can be reused by AUT^17,27,39^.

### Competition between microbial families

**Figure 7:**
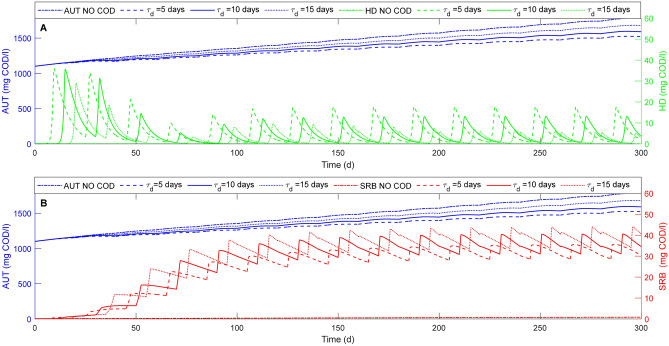
Time evolution of heterotrophic species (SRB and HD) concentrations and autotrophic biomass concentration for an SBR cycle duration of 20 days with no COD (.-) and stoichiometric COD injection at three different ${\uptau }_{{\text{d}}}$ of 5(---), 10(-) and 15(…) days.

The results obtained in terms of removal efficiency of nitrogenous compounds and sulfate, are reflected in the growth trend of the microbial families involved in the process. The results in terms of HD, AUT and SRB concentrations are reported in Fig. 7 for different $\tau_{d}$ values and a stoichiometric COD addition.

The growth of autotrophic denitrifiers is mainly observed after the first three SBR cycles leading to a higher sulfate production which is related to an increase of the SRB growth. Thus, the value of $\tau_{d}$ has a strong influence on the evolution of the concentration of the three microbial families (Fig. 7). An increase of $\tau_{d}$ results in a longer first reaction period where the main process is SdAD, while a lower $\tau_{d}$ leads to an increase of the HD growth. This is enhanced by the higher nitrate and nitrite concentrations available (Fig. 6C). At the same time, a longer $\tau_{d}$ leads to a higher sulfate accumulation (Fig. 6E,D), which stimulates the growth of SRB. Moreover, the growth of the heterotrophic biomasses (both HD and SRB) is higher when a higher COD amount is provided at lower $\tau_{d}$ values, as it is possible to observe by the fast consumption of sulfate and nitrate in Fig. 6B,C . Despite an initial increase of HD concentration observed for all $\tau_{d}$ values (Fig. 7A) during the first SBR cycles, the lowest value of $\tau_{d}$ leads to the highest concentration of HD, which remains lower than the SRB. The $\tau_{d}$ variation affects the two different reaction phases and has an impact on the competition between the different microbial families involved except for HYD (data not shown), whose growth only depends on the initial concentration of both biomass and elemental sulfur.

The results obtained with this model in terms of microbial families profiles are consistent with those achieved experimentally, which show that the heterotrophic denitrifying bacteria never prevail over autotrophic denitrifiers when a proper acclimatation of autotrophs on elemental sulfur is performed^5^. Indeed, the prevalent microbial family is represented by the autotrophic denitrifiers in each simulated scenario (Fig. 7). As discussed before, heterotrophic denitrification is faster than SdAD, but the two processes do not significantly affect each other^18,41^. As reported by Liu et al.^25^, an increase of the COD injected leads to an increase of the concentration of HD. Furthermore, the low concentration of HD can be also related to the high solid retention time achievable in a SBR, which facilitates the retention of slow-growing bacteria such as AUT.

### SMP evolution

**Figure 8:**
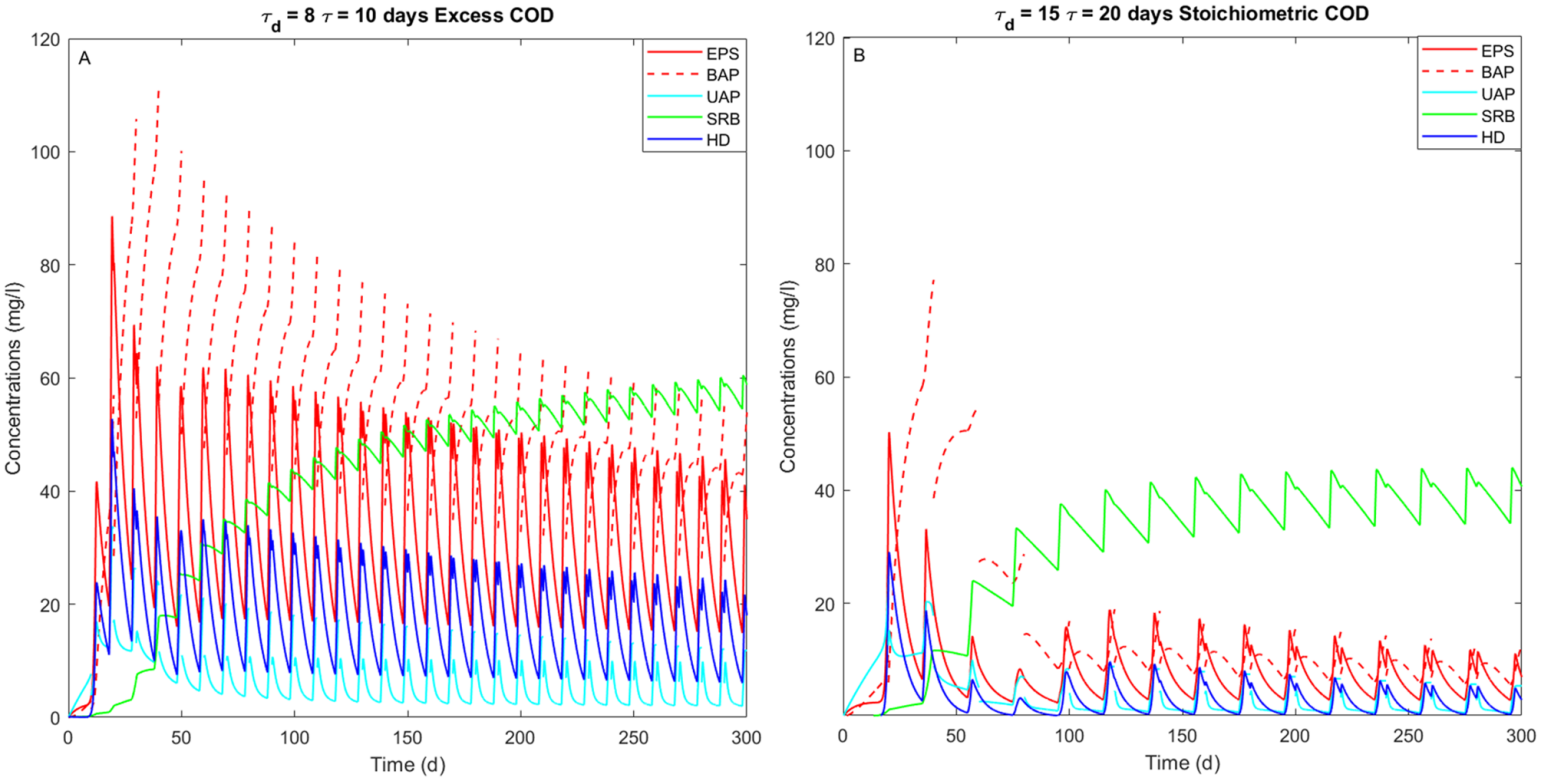
SMP production and concentration of heterotrophic families (i.e. HD and SRB) overtime in two different cases: (**A**) excess COD at $\tau_{d}$ = 8 days and $\tau$ = 10 days; (**B**) stoichiometric COD at $\tau_{d}$ = 15 days and $\tau$ = 20 days. The solid dark blue line represents the heterotrophic denitrifiers (HD), while the solid green line indicates the sulfate-reducing bacteria (SRB). The solid red line indicates the EPS, which allows the production of BAP (red dashed line) after hydrolysis. The solid light blue line represents the UAP.

The simultaneous presence of different active microbial families leads to an increased SMP production compared to that obtained when only autotrophic denitrifiers are the main bacteria involved (Fig. 5). In particular, the higher concentration of heterotrophic families, both HD and SRB, in presence of external COD implies higher EPS, BAP and UAP concentrations (Fig. 8).

The highest SMP concentrations are obtained in the presence of a COD injection in excess to the theoretical value required for complete sulfate reduction (Fig. 8A). Moreover, as it possible to observe from Fig. 6B (excess COD at $\tau_{d}$ = 8 days, $\tau$ = 10 days,) and 6E (stoichiometric COD at $\tau_{d}$ = 15 days $,\tau$ = 20 days), the nitrate and sulfate removal efficiencies follow a similar trend in both simulations, but different SMP amounts are produced (Fig. 8) The results obtained are related to the different families involved in the two cases. Indeed, when the denitrification is mostly conducted by autotrophs (Fig. 6E), as indicated by the higher sulfate production and the lower HD concentration, lower SMP amounts are produced (Fig. 8B). This is because the activity of heterotrophs result in a higher SMP production, also enhanced by the COD addition.

**Figure 9:**
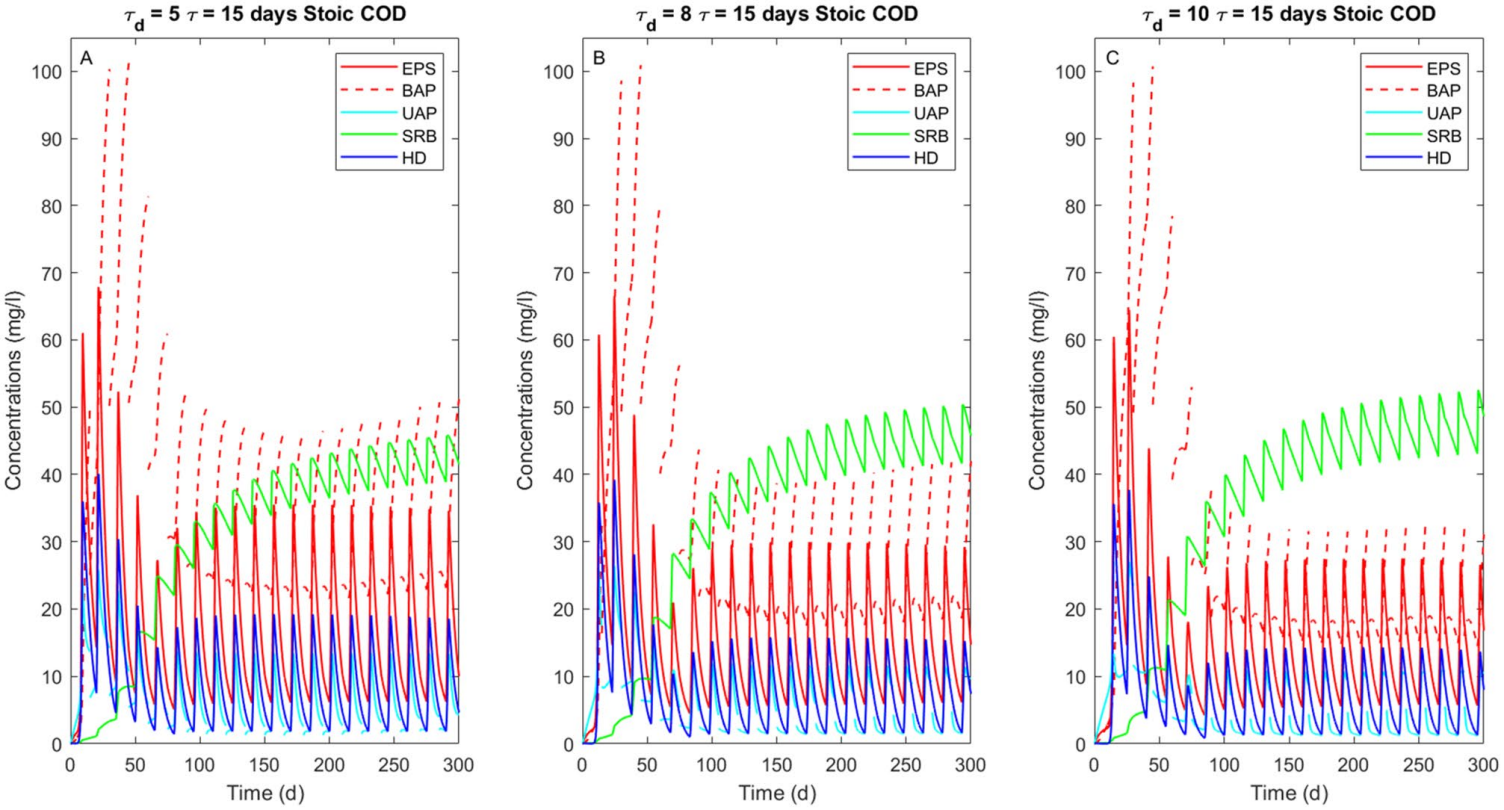
SMP production and concentration of heterotrophic families (i.e. HD and SRB) overtime increasing ${\uptau }_{{\text{d}}}$ from 5 (**A**) to 8 (**B**) and 10 days (**C**) with an injection of stoichiometric COD. The solid dark blue line represents the heterotrophic denitrifiers (HD), while the solid green line indicates the sulfate-reducing bacteria (SRB). The solid red line indicates the EPS, which allows the production of BAP (red dashed line) after hydrolysis. The solid light blue line represents the UAP.

The evaluation of the SMP associated with the growth of the microbial families could be also used to control and prevent the undesirable COD products, which is normally considered as a secondary pollution in the effluent^17^. With respect to the case without COD addition (Fig. 5), the injection of a stoichiometric COD amount promotes the growth of both heterotrophic families (i.e. SRB and HD) and consequently an increased production of UAP and EPS (Fig. 9). About the formation and use of SMP, UAP and BAP, these are more pronounced in the cases of short cycle durations and low $\tau_{d}$, since the growth of HD is enhanced by the COD concentration (Fig. 8A). This observation is in line with experimental evidences from Tian et al. (2011)^42^, who evaluated the concentration of SMP produced during the simultaneous growth of heterotrophs and autotrophs where a higher heterotrophic growth was associated with a higher SMP production.

Figure 9 shows that the day of COD injection affects the correlation between SRB and SMP. In particular, a higher SMP concentration is related to a lower SRB presence. Up to now, no experimental evidence shows SMP production and uptake by both the heterotrophic families involved in this model. Nonetheless, Asik et al.^43^ and Ucar et al.^10^ reported that a SdAD effluent requires a higher transmembrane pressure to be separated from a nanofiltration process at increasing sulfate amounts. In those cases the increase in sulfate production was linked to higher amount of nitrate injected, associated to shorter retention time. The authors attributed this to the high production of SMP, which were not consumed by the heterotrophic families present in the system probably because part of the SMP requires longer time to be bioavailable.

## Conclusion

In this work, we presented a model investigating the dynamics of SdAD as the main process occurring in the presence of elemental sulfur as inorganic electron donor. The model also takes into account the growth of two heterotrophic families, i.e. HD and SRB, naturally growing in sulfur-governed autotrophic systems. Numerical simulations investigated to which extent the heterotrophic denitrification and sulfate reduction, promoted by COD addition and SMP production, affect autotrophic denitrification performance. We observed that the growth of the two heterotrophic families is favored by SMP production and mainly COD when an external carbon source is provided. The results obtained are in line with the intended objectives: (1) the concentration of sulfate in the effluent is lower in the scenarios where the COD injection occurs, even for low values of $\tau_{d}$; (2) the simultaneous activity of both heterotrophic biomasses leads to a better performance of the process; (3) SRB can also grow also in the absence of an external carbon addition.

The model reproduces with a good approximation the experimental observations in terms of microbial families and process performance, representing a tool capable of responding to different needs that are mainly represented by:

- Nitrate, nitrite and sulfate effluent concentrations;
- The simultaneous growth of three different microbial families;
- The competition between the two heterotrophic families involved;
- The effects of SMP on the heterotrophic growth;
- The influence of the reaction period;
- The influence of COD addition on the efficiency of SdAD, heterotrophic denitrification and sulfate reduction.

Future work will be necessary to experimentally calibrate the kinetic parameters of the model, in particular those associated with the production of the different SMP related to the activity of autotrophic denitrifiers using elemental sulfur. This might require the development of ad-hoc experimental activities which elucidate the SMP production during SdAD process as none of the previous studies have evaluated the competition between the three microbial families here taken into account on the SMP. Furthermore, the model needs to be also validated under different operating conditions, for instance also measuring the N2O emission, which can represent a shortcoming of SdAD.

## Supplementary Information

Supplementary Information.
