# Supplementary material for: Modelling the effect of SMP production and external carbon addition on S-driven autotrophic denitrification
Source: Sci Rep. 2022 Apr 29;12:7008. doi: 10.1038/s41598-022-10944-z (PMC9054823; doi:10.1038/s41598-022-10944-z)
Supplement: Supplementary file 1 — Supplementary Information. [file 41598_2022_10944_MOESM1_ESM.docx]

# **Modelling the effect of SMP production and external carbon addition on S-driven autotrophic denitrification**

Grazia Guerriero^1^*, Maria Rosaria Mattei ^1^, Stefano Papirio^2^, Giovanni Esposito^2^, Luigi Frunzo^1^

^1^ Department of Mathematics and Applications “R. Caccioppoli”, via Cintia, Monte S. Angelo, 80126, Naples, Italy

^2^ Department of Civil, Architectural and Environmental Engineering, University of Naples Federico II, via Claudio 21, 80125, Naples, Italy

* Corresponding author: Grazia Guerriero ([grazia.guerriero@unina.it](mailto:grazia.guerriero@unina.it))

*Table S.1 Matrix for the stoichiometric values referred to biomasses.*

| REACTION TYPE | $\boldsymbol{X}_{\boldsymbol{HID}}$ | $\boldsymbol{X}_{\boldsymbol{AUT}}$ | $\boldsymbol{X}_{\boldsymbol{STOB}}$ | $\boldsymbol{X}_{\mathbf{EPS}}$ | $\boldsymbol{X}_{\boldsymbol{HD}}$ | $\boldsymbol{X}_{\mathbf{SRB}}$ | $\boldsymbol{X}_{\mathbf{I}}$ |
| --- | --- | --- | --- | --- | --- | --- | --- |
| $\mathbf{1. Hydrolysis of S}_{\mathbf{0}}$ |  |  |  |  |  |  |  |
| $\mathbf{2. Autotrophic denitrification}$  $\mathbf{N}\mathbf{O}_{\mathbf{3}}^{\mathbf{-}}\boldsymbol{\to N}\mathbf{O}_{\mathbf{2}}^{\mathbf{-}}$ |  | $1-f_{EPS,AUT}-f_{UAP,AUT}$ |  | $f_{EPS,AUT}$ |  |  |  |
| $\mathbf{3.Autotrophic denitrification}$  $\mathbf{N}\mathbf{O}_{\mathbf{2}}^{\mathbf{-}}\boldsymbol{\to}\mathbf{N}_{\mathbf{2}}$ |  | $1-f_{EPS,AUT}-f_{UAP,AUT}$ |  | $f_{EPS,AUT}$ |  |  |  |
| $\mathbf{4.Decay}\mathbf{X}_{\mathbf{HID}}$ | $-1$ |  | $1-f_{I}$ |  |  |  | $f_{I}$ |
| $\mathbf{5.Decay}\mathbf{X}_{\mathbf{AUT}}$ |  | $-1$ | $1-f_{I}-f_{\mathrm{BAP}}$ |  |  |  | $f_{I}$ |
| $\mathbf{6.Hydrolysis}$ of $\mathbf{X}_{\mathbf{STOB}}$ |  |  | $-1$ |  |  |  |  |
| $\mathbf{7.Release of}\mathbf{X}_{\mathbf{EPS}}$ |  |  |  | $-1$ |  |  |  |
| $\mathbf{8.Growth of}\mathbf{X}_{\mathbf{HD}}\mathbf{on}\mathbf{S}_{\mathbf{COD}}\mathbf{and}\mathbf{S}_{\mathbf{NO2}}$ |  |  |  | $\frac{k_{\mathrm{EPS}}}{y_{H,COD}}$ | $1-\frac{k_{\mathrm{UAP}}}{y_{H,COD}}-\frac{k_{\mathrm{EPS}}}{y_{H,COD}}$ |  |  |
| $\mathbf{9.Growth of}\mathbf{X}_{\mathbf{HD}}\mathbf{on}\mathbf{S}_{\mathbf{COD}}\mathbf{and}\mathbf{S}_{\mathbf{NO3}}$ |  |  |  | $\frac{k_{\mathrm{EPS}}}{y_{H,COD}}$ | $1-\frac{k_{\mathrm{UAP}}}{y_{H,COD}}-\frac{k_{\mathrm{EPS}}}{y_{H,COD}}$ |  |  |
| $\mathbf{10.Growth of}\mathbf{X}_{\mathbf{HD}}\mathbf{on}\mathbf{S}_{\mathbf{UAP}}\mathbf{and}\mathbf{S}_{\mathbf{NO2}}$ |  |  |  |  | $1$ |  |  |
| $\mathbf{11.Growth of}\mathbf{X}_{\mathbf{HD}}\mathbf{on}\mathbf{S}_{\mathbf{UAP}}\mathbf{and}\mathbf{S}_{\mathbf{NO3}}$ |  |  |  |  | $1$ |  |  |
| $\mathbf{12.Growth of}\mathbf{X}_{\mathbf{HD}}\mathbf{on}\mathbf{S}_{\mathbf{BAP}}\mathbf{and}\mathbf{S}_{\mathbf{NO2}}$ |  |  |  |  | $1$ |  |  |
| $\mathbf{13.Growth of}\mathbf{X}_{\mathbf{HD}}\mathbf{on}\mathbf{S}_{\mathbf{BAP}}\mathbf{and}\mathbf{S}_{\mathbf{NO3}}$ |  |  |  |  | $1$ |  |  |
| $\mathbf{14.Decay of}\mathbf{X}_{\mathbf{HD}}$ |  |  | $1-f_{I}-f_{\mathrm{BAP}}$ |  | $-1$ |  | $f_{I}$ |
| $\mathbf{15.Growth of}\mathbf{X}_{\mathbf{SRB}}\mathbf{on}\mathbf{S}_{\mathbf{COD}}$ |  |  |  | $f_{EPS,SRB}$ |  | $1-f_{EPS,SRB}-f_{UAP,SRB}$ |  |
| $\mathbf{16.Decay of}\mathbf{X}_{\mathbf{SRB}}$ |  |  | $1-f_{I}-f_{\mathrm{BAP}}$ |  |  | $-1$ | $f_{I}$ |
| $\mathbf{17.Growth of}\mathbf{X}_{\mathbf{SRB}}\mathbf{on}\mathbf{S}_{\mathbf{UAP}}$ |  |  |  |  |  | 1 |  |
| $\mathbf{18.Growth of}\mathbf{X}_{\mathbf{SRB}}\mathbf{on}\mathbf{S}_{\mathbf{BAP}}$ |  |  |  |  |  | 1 |  |

*Table S.2 Matrix for the stoichiometric values referred to Substrates.*

| REACTION TYPE | $\boldsymbol{S}_{\boldsymbol{S}_{\mathbf{0}}}$ | $\boldsymbol{S}_{\boldsymbol{S}_{\boldsymbol{b}}}$ | $\boldsymbol{S}_{\boldsymbol{N}\boldsymbol{O}_{\mathbf{3}}^{\mathbf{-}}}$ | $\boldsymbol{S}_{\boldsymbol{N}\boldsymbol{O}_{\mathbf{2}}^{\mathbf{-}}}$ | $\boldsymbol{S}_{\mathbf{N}_{\mathbf{2}}}$ | $\boldsymbol{S}_{\boldsymbol{S}\boldsymbol{O}_{\boldsymbol{4}}^{\boldsymbol{2-}}}$ | $\boldsymbol{S}_{\boldsymbol{UAP}}$ | $\boldsymbol{S}_{\boldsymbol{BAP}}$ | $\boldsymbol{S}_{\mathbf{COD}}$ |
| --- | --- | --- | --- | --- | --- | --- | --- | --- | --- |
| $\mathbf{1. Hydrolysis of S}_{\mathbf{0}}$ | $-1$ | $1$ |  |  |  |  |  |  |  |
| $\mathbf{2. Autotrophic denitrification}$  $\mathbf{N}\mathbf{O}_{\mathbf{3}}^{\mathbf{-}}\boldsymbol{\to N}\mathbf{O}_{\mathbf{2}}^{\mathbf{-}}$ |  | $-\frac{b_{1}}{y_{AUT,NO_{3}^{-}}}$ | $-\frac{1}{y_{AUT,NO_{3}^{-}}}$ | $\frac{1}{y_{AUT,NO_{3}^{-}}}$ |  | $\frac{b_{1}}{y_{AUT,NO_{3}^{-}}}$ | $f_{UAP,AUT}$ |  |  |
| $\mathbf{3.Autotrophic denitrification}$  $\mathbf{N}\mathbf{O}_{\mathbf{2}}^{\mathbf{-}}\boldsymbol{\to}\mathbf{N}_{\mathbf{2}}$ |  | $-\frac{b_{2}}{y_{AUT,NO_{2}^{-}}}$ |  | $-\frac{1}{y_{AUT,NO_{2}^{-}}}$ | $\frac{1}{y_{AUT,NO_{2}^{-}}}$ | $\frac{b_{2}}{y_{AUT,NO_{2}^{-}}}$ | $f_{UAP,AUT}$ |  |  |
| $\mathbf{4.Decay}\mathbf{X}_{\mathbf{HID}}$ |  |  |  |  |  |  |  | $f_{\mathrm{BAP}}$ |  |
| $\mathbf{5.Decay}\mathbf{X}_{\mathbf{AUT}}$ |  |  |  |  |  |  |  | $f_{\mathrm{BAP}}$ |  |
| $\mathbf{6.Hydrolysis}$ of $\mathbf{X}_{\mathbf{STOB}}$ |  |  |  |  |  |  |  |  | $1$ |
| $\mathbf{7.Release of}\mathbf{X}_{\mathbf{EPS}}$ |  |  |  |  |  |  |  | $1$ |  |
| $\mathbf{8.Growth of}\mathbf{X}_{\mathbf{HD}}\mathbf{on}\mathbf{S}_{\mathbf{COD}}\mathbf{and}\mathbf{S}_{\mathbf{NO2}}$ |  |  |  | $-\frac{\left( 1-y_{H,COD} \right)\left( 1-k_{\mathrm{UAP}}-k_{\mathrm{EPS}} \right)}{1,71y_{H,COD}}$ | $\frac{\left( 1-y_{H,COD} \right)\left( 1-k_{\mathrm{UAP}}-k_{\mathrm{EPS}} \right)}{1,71y_{H,COD}}$ |  | $\frac{k_{\mathrm{UAP}}}{y_{H,COD}}$ |  | $-\frac{1}{y_{H,COD}}$ |
| $\mathbf{9.Growth of}\mathbf{X}_{\mathbf{HD}}\mathbf{on}\mathbf{S}_{\mathbf{COD}}\mathbf{and}\mathbf{S}_{\mathbf{NO3}}$ |  |  | $-\frac{\left( 1-y_{H,COD} \right)\left( 1-k_{\mathrm{UAP}}-k_{\mathrm{EPS}} \right)}{2,86y_{H,COD}}$ |  | $\frac{\left( 1-y_{H,COD} \right)\left( 1-k_{\mathrm{UAP}}-k_{\mathrm{EPS}} \right)}{2,86y_{H,COD}}$ |  | $\frac{k_{\mathrm{UAP}}}{y_{H,COD}}$ |  | $-\frac{1}{y_{H,COD}}$ |
| $\mathbf{10.Growth of}\mathbf{X}_{\mathbf{HD}}\mathbf{on}\mathbf{S}_{\mathbf{UAP}}\mathbf{and}\mathbf{S}_{\mathbf{NO2}}$ |  |  |  | $-\frac{\left( 1-y_{H,UAP} \right)}{1,71y_{H,UAP}}$ | $\frac{\left( 1-y_{H,UAP} \right)}{1,71y_{H,UAP}}$ |  | $-\frac{1}{y_{H,UAP}}$ |  |  |
| $\mathbf{11.Growth of}\mathbf{X}_{\mathbf{HD}}\mathbf{on}\mathbf{S}_{\mathbf{UAP}}\mathbf{and}\mathbf{S}_{\mathbf{NO3}}$ |  |  | $-\frac{\left( 1-y_{H,UAP} \right)}{2,86y_{H,UAP}}$ |  | $\frac{\left( 1-y_{H,UAP} \right)}{2,86y_{H,UAP}}$ |  | $-\frac{1}{y_{H,UAP}}$ |  |  |
| $\mathbf{12.Growth of}\mathbf{X}_{\mathbf{HD}}\mathbf{on}\mathbf{S}_{\mathbf{BAP}}\mathbf{and}\mathbf{S}_{\mathbf{NO2}}$ |  |  |  | $-\frac{\left( 1-y_{H,BAP} \right)}{1,71y_{H,BAP}}$ | $\frac{\left( 1-y_{H,BAP} \right)}{1,71y_{H,BAP}}$ |  |  | $-\frac{1}{y_{H-BAP}}$ |  |
| $\mathbf{13.Growth of}\mathbf{X}_{\mathbf{HD}}\mathbf{on}\mathbf{S}_{\mathbf{BAP}}\mathbf{and}\mathbf{S}_{\mathbf{NO3}}$ |  |  | $-\frac{\left( 1-y_{H,BAP} \right)}{2,86y_{H,BAP}}$ |  | $\frac{\left( 1-y_{H,BAP} \right)}{2,86y_{H,BAP}}$ |  |  | $-\frac{1}{y_{H-BAP}}$ |  |
| $\mathbf{14.Decay of}\mathbf{X}_{\mathbf{HD}}$ |  |  |  |  |  |  |  | $f_{\mathrm{BAP}}$ |  |
| $\mathbf{15.Growth of}\mathbf{X}_{\mathbf{SRB}}\mathbf{on}\mathbf{S}_{\mathbf{COD}}$ |  | $\frac{1}{2}\left( \frac{1-y_{\mathrm{SRB}}}{y_{\mathrm{SRB}}} \right)$ |  |  |  | $-\frac{1}{2}\left( \frac{1-y_{\mathrm{SRB}}}{y_{\mathrm{SRB}}} \right)$ | $f_{UAP,SRB}$ |  | $-\frac{1}{y_{\mathrm{SRB}}}$ |
| $\mathbf{16.Decay of}\mathbf{X}_{\mathbf{SRB}}$ |  |  |  |  |  |  |  | $f_{\mathrm{BAP}}$ |  |
| $\mathbf{17.Growth of}\mathbf{X}_{\mathbf{SRB}}\mathbf{on}\mathbf{S}_{\mathbf{UAP}}$ |  | $\frac{1}{2}\left( \frac{1-y_{\mathrm{SRB}}}{y_{\mathrm{SRB}}} \right)$ |  |  |  | $-\frac{1}{2}\left( \frac{1-y_{\mathrm{SRB}}}{y_{\mathrm{SRB}}} \right)$ | $-\frac{1}{y_{\mathrm{SRB}}}$ |  |  |
| $\mathbf{18.Growth of}\mathbf{X}_{\mathbf{SRB}}\mathbf{on}\mathbf{S}_{\mathbf{BAP}}$ |  | $\frac{1}{2}\left( \frac{1-y_{\mathrm{SRB}}}{y_{\mathrm{SRB}}} \right)$ |  |  |  | $-\frac{1}{2}\left( \frac{1-y_{\mathrm{SRB}}}{y_{\mathrm{SRB}}} \right)$ |  | $-y_{\mathrm{SRB}}$ |  |

*Table S.3. Stoichiometric constant values*

|  | **Description** | **Value** | **Unit** | **Source** |
| --- | --- | --- | --- | --- |
| $\boldsymbol{f}_{\boldsymbol{EPS,AUT}}$ | Fraction of $X_{\mathrm{EPS}}$ for $X_{\mathrm{AUT}}$biomass growth | 0.09 | mg COD/mg N | ^1^ |
| $\boldsymbol{f}_{\boldsymbol{UAP,AUT}}$ | Fraction of $S_{\mathrm{UAP}}$ for$X_{\mathrm{AUT}}$ biomass growth | 0.14 | mg COD/mg N | ^1^ |
| $\boldsymbol{f}_{\boldsymbol{BAP}}$ | Fraction of $S_{\mathrm{BAP}}$ forbiomass growth | 0.0215 | mg COD/mg COD | ^2^ |
| $\boldsymbol{f}_{\boldsymbol{I}}$ | Fraction of $X_{I}$ in biomass decay | 0.08 | mg COD/mg COD | ^3^ |
| $\boldsymbol{k}_{\boldsymbol{EPS}}$ | Yield coefficient for $X_{\mathrm{EPS}}$ for $X_{\mathrm{HD}}$ | 0.14 | mg COD/mg COD | ^4^ |
| $\boldsymbol{k}_{\boldsymbol{UAP}}$ | Yield coefficient for $S_{\mathrm{UAP}}$ for $X_{\mathrm{HD}}$ | 0.09 | mg COD/mg COD | ^4^ |
| $\boldsymbol{b}_{\boldsymbol{1}}$ | $S_{S_{b}}$ to $S_{NO_{3}^{-}}$ stoichiometric ratio | 1.2 | mg S/ mg N | ^5^ |
| $\boldsymbol{b}_{\boldsymbol{2}}$ | $S_{S_{b}}$ to $S_{NO_{2}^{-}}$ stoichiometric ratio | 0.55 | mg S/ mg N | ^5^ |
| $\boldsymbol{y}_{\boldsymbol{AUT,N}\boldsymbol{O}_{\boldsymbol{3}}^{\boldsymbol{-}}}$ | Yield coefficient for $X_{\mathrm{AUT}}$ on $S_{NO_{3}^{-}}$ | 0.37 | mg COD/mg N | ^6^ |
| $\boldsymbol{y}_{\boldsymbol{AUT,N}\boldsymbol{O}_{\boldsymbol{2}}^{\boldsymbol{-}}}$ | Yield coefficient for $X_{\mathrm{AUT}}$ on $S_{NO_{2}^{-}}$ | 0.414 | mg COD/mg N | ^6^ |
| $\boldsymbol{f}_{\boldsymbol{EPS,SRB}}$ | Fraction of $X_{\mathrm{EPS}}$ for $X_{\mathrm{SRB}}$ biomass growth | 0.9 | mg COD/mg COD | assumed |
| $\boldsymbol{f}_{\boldsymbol{UAP}\boldsymbol{,SRB}}$ | Fraction of $S_{\mathrm{UAP}}$ for $X_{\mathrm{SRB}}$ biomass growth | 0.14 | mg COD/mg COD | assumed |
| $\boldsymbol{y}_{\boldsymbol{H,COD}}$ | Yield coefficient for $X_{\mathrm{HD}}$ on $S_{\mathrm{COD}}$ | 0.34 | mg COD/mg COD | ^4^ |
| $\boldsymbol{y}_{\boldsymbol{H,UAP}}$ | Yield coefficient for $X_{\mathrm{HD}}$ on $S_{\mathrm{UAP}}$ | 0.45 | mg COD/mg COD | ^4^ |
| $\boldsymbol{y}_{\boldsymbol{H,BAP}}$ | Yield coefficient for $X_{\mathrm{HD}}$ on $S_{\mathrm{BAP}}$ | 0.45 | mg COD/mg COD | ^4^ |
| $\boldsymbol{y}_{\boldsymbol{SRB}}$ | Yield coefficient for $X_{\mathrm{SRB}}$ | 0.0568 | mg COD/mg COD | ^7^ |

*Table S.4. Reaction terms*

| *j- process* |  | *Process rate (*$\boldsymbol{\rho}_{\boldsymbol{j}}$*)* |
| --- | --- | --- |
| 1 | Hydrolysis of elemental sulfur  $S_{0} \to S_{\mathrm{bio}}$ | $K_{0}\cdot k_{1}\frac{S_{S^{0}}}{\frac{\kappa_{1}}{a^{*}}+S_{S^{0}}}X_{HID}$ |
| 2 | Autotrophic denitrification$NO_{3}^{-}\to NO_{2}^{-}$ | $\mu_{S_{b},NO_{3}^{-}}^{max}\frac{S_{S_{b}}}{k_{AUT{,S}_{b}}+S_{NO_{2}^{-}}}\cdot\frac{S_{NO_{3}^{-}}}{k_{AUT,NO_{3}^{-}}+S_{NO_{3}^{-}}}\cdot\frac{S_{NO_{3}^{-}}}{S_{NO_{3}^{-}}+S_{NO_{2}^{-}}}\cdot X_{AUT}$ |
| 3 | $Autotrophic denitrification$  $NO_{2}^{-} \to N_{2}$ | $\mu_{S_{b},NO_{2}^{-}}^{max}\frac{S_{S_{b}}}{k_{AUT{,S}_{b}}+S_{S_{b}}}\cdot\frac{S_{NO_{2}^{-}}}{k_{AUT,NO_{2}^{-}}+S_{NO_{2}^{-}}}\cdot\frac{S_{NO_{2}^{-}}}{S_{NO_{3}^{-}}+S_{NO_{2}^{-}}}\cdot X_{AUT}$ |
| 4 | Decay of HYD | $k_{d,HID}\cdot X_{HID}$ |
| 5 | Decay of AUT | $k_{d,AUT}\cdot X_{AUT}$ |
| 6 | $Hydrolysis of organic carbon$  $X_{\mathrm{STO}} \to S_{\mathrm{COD}}$ | $k_{HID,STOB}\cdot\frac{X_{STO}/X_{HD}}{k_{STOB,COD}+X_{STO}/X_{HD}}X_{HD}$ |
| 7 | $Release of EPS$  $X_{\mathrm{EPS}} \to S_{\mathrm{BAP}}$ | $K_{HID,EPS}\cdot X_{EPS}$ |
| 8 | $Growth of X_{\mathrm{HD}}\mathrm{on}S_{S}\mathrm{and}S_{NO2}$ | $\mu_{HD,COD}\cdot\eta_{ox}\cdot\frac{S_{COD}}{k_{COD,HD}+S_{COD}}\cdot\frac{S_{NO_{2}^{-}}}{k_{NO_{2}}^{HD}+S_{NO_{2}^{-}}}\cdot\frac{S_{NO_{2}^{-}}}{S_{NO_{2}^{-}}+S_{NO_{3}^{-}}}\cdot\frac{S_{COD}}{S_{UAP}+S_{BAP}+S_{COD}}\cdot X_{HD}$ |
| 9 | $Growth of X_{\mathrm{HD}}\mathrm{on}S_{S}\mathrm{and}S_{NO3}$ | $\mu_{HD,COD}\cdot\eta_{ox}\cdot\frac{S_{COD}}{k_{COD,HD}+S_{COD}}\cdot\frac{S_{NO_{3}^{-}}}{k_{NO_{3}}^{HD}+S_{NO_{3}^{-}}}\cdot\frac{S_{NO_{3}^{-}}}{S_{NO_{2}^{-}}+S_{NO_{3}^{-}}}\cdot\frac{S_{COD}}{S_{UAP}+S_{BAP}+S_{COD}}\cdot X_{HD}$ |
| 10 | $Growth of X_{\mathrm{HD}}\mathrm{on}S_{\mathrm{UAP}}\mathrm{and}S_{NO2}$ | $\mu_{HD,UAP}\cdot\eta_{OX}\cdot\frac{S_{UAP}}{k_{UAP}+S_{UAP}}\cdot\frac{S_{NO_{2}^{-}}}{k_{NO_{2}}^{HD}+S_{NO_{2}^{-}}}\cdot\frac{S_{NO_{2}^{-}}}{S_{NO_{2}^{-}}+S_{NO_{3}^{-}}}\cdot\frac{S_{UAP}}{S_{UAP}+S_{BAP}+ S_{COD}}\cdot X_{HD}$ |
| 11 | $Growth of X_{\mathrm{HD}}\mathrm{on}S_{\mathrm{UAP}}\mathrm{and}S_{NO3}$ | $\mu_{HD,UAP}\cdot\eta_{OX}\cdot\frac{S_{UAP}}{k_{UAP}+S_{UAP}}\cdot\frac{S_{NO_{3}^{-}}}{k_{NO_{3}}^{HD}+S_{NO_{3}^{-}}}\cdot\frac{S_{NO_{3}^{-}}}{S_{NO_{2}^{-}}+S_{NO_{3}^{-}}}\cdot\frac{S_{UAP}}{S_{UAP}+S_{BAP}+S_{COD}}\cdot X_{HD}$ |
| 12 | $Growth of X_{\mathrm{HD}}\mathrm{on}S_{\mathrm{BAP}}\mathrm{and}S_{NO2}$ | $\mu_{HD,BAP}\cdot\eta_{OX}\cdot\frac{S_{BAP}}{k_{BAP}+S_{BAP}}\cdot\frac{S_{NO_{2}^{-}}}{k_{NO_{2}}^{HD}+ S_{NO_{2}^{-}}}\cdot\frac{S_{NO_{2}^{-}}}{S_{NO_{2}^{-}}+ S_{NO_{3}^{-}}}\cdot\frac{S_{BAP}}{S_{UAP}+S_{BAP}+S_{COD}}\cdot X_{HD}$ |
| 13 | $Growth of X_{\mathrm{HD}}\mathrm{on}S_{\mathrm{BAP}}\mathrm{and}S_{NO3}$ | $\mu_{HD,BAP}\cdot\eta_{OX}\cdot\frac{S_{BAP}}{k_{BAP}+S_{BAP}}\cdot\frac{S_{NO_{3}^{-}}}{k_{NO_{3}}^{HD}+S_{NO_{3}^{-}}}\cdot\frac{S_{NO_{3}^{-}}}{S_{NO_{2}^{-}}+S_{NO_{3}^{-}}}\cdot\frac{S_{BAP}}{S_{UAP}+S_{BAP}+S_{COD}}\cdot X_{HD}$ |
| 14 | $Decay of X_{\mathrm{HD}}$ | $k_{d,HD}\cdot X_{HD}$ |
| 15 | $Growth of X_{\mathrm{SRB}}\mathrm{on}S_{S}$ | $\mu_{SRB}\cdot\frac{S_{COD}}{k_{COD,SRB}+S_{COD}}\cdot\frac{S_{SO_{4}^{2-}}}{k_{SRB,SO_{4}}+S_{SO_{4}^{2-}}}\cdot\frac{S_{COD}}{S_{UAP}+S_{BAP}+S_{COD}}\cdot X_{SRB}$ |
| 16 | $Growth of X_{\mathrm{SRB}} on UAP$ | $\mu_{SRB}\cdot\frac{\mu_{HD,UAP}}{\mu_{HD,COD}}\cdot\frac{S_{UAP}}{k_{COD,SRB}+S_{UAP}}\cdot\frac{S_{SO_{4}^{2-}}}{k_{SRB,SO_{4}}+S_{SO_{4}^{2-}}}\cdot\frac{S_{UAP}}{S_{UAP}+S_{BAP}+S_{COD}}\cdot X_{SRB}$ |
| 17 | $Growth of X_{\mathrm{SRB}} on BAP$ | $\mu_{SRB}\cdot\frac{\mu_{HD,BAP}}{\mu_{HD,COD}}\cdot\frac{S_{BAP}}{k_{COD,SRB}+S_{BAP}}\cdot\frac{S_{SO_{4}^{2-}}}{k_{SRB,SO_{4}}+S_{SO_{4}^{2-}}}\cdot\frac{S_{BAP}}{S_{UAP}+S_{BAP}+S_{COD}}\cdot X_{SRB}$ |
| 18 | $Decay of X_{\mathrm{SRB}}$ | $k_{d,SRB}\cdot X_{SRB}$ |

*Table S.5. Kinetic constant values*

|  | **Description** | **Value** | **Unit** | **Ref.** |
| --- | --- | --- | --- | --- |
| $\boldsymbol{K}_{\boldsymbol{0}}$ | Efficiency growth coefficient for $X_{\mathrm{HID}}$ | 0.1 | mg COD/ mg S | ^8^ |
| $\boldsymbol{\mu}_{\boldsymbol{S}_{\boldsymbol{b}}\boldsymbol{,}\boldsymbol{N}\boldsymbol{O}_{\boldsymbol{3}}^{\boldsymbol{-}}}^{\boldsymbol{max}}$ | Maximum growth rate for $X_{\mathrm{AUT}}$ on$S_{NO_{3}^{-}}$ | 0.0067 | $d^{-1}$ | ^8^ |
| $\boldsymbol{\mu}_{\boldsymbol{S}_{\boldsymbol{b}}\boldsymbol{,}\boldsymbol{N}\boldsymbol{O}_{\boldsymbol{2}}^{\boldsymbol{-}}}^{\boldsymbol{max}}$ | Maximum growth rate for $X_{\mathrm{AUT}}$ on $S_{NO_{2}^{-}}$ | 0.0058 | $d^{-1}$ | ^8^ |
| $\boldsymbol{k}_{\boldsymbol{AUT}\boldsymbol{,S}_{\boldsymbol{b}}}$ | Half-saturation constant for $S_{S_{b}}$ | 0.215 | mg S/l | ^9^ |
| $\boldsymbol{k}_{\boldsymbol{AUT}\boldsymbol{,}\boldsymbol{N}\boldsymbol{O}_{\boldsymbol{3}}^{\boldsymbol{-}}}$ | Half-saturation constant for $S_{NO_{3}^{-}}$ | 36 | mg N/l | ^8^ |
| $\boldsymbol{k}_{\boldsymbol{AUT}\boldsymbol{,}\boldsymbol{N}\boldsymbol{O}_{\boldsymbol{2}}^{\boldsymbol{-}}}$ | Half-saturation constant for $S_{NO_{3}^{-}}$ | 40 | mg N/l | ^6^ |
| $\boldsymbol{k}_{\boldsymbol{1}}$ | Hydrolysis kinetic constant | 0.081 | mg S/ mg COD $\cdot$d | ^8^ |
| $\boldsymbol{\kappa}_{\boldsymbol{1}}$ | Volume specific half-saturation constant for $S_{S^{0}}$ | 5.1 | 1/dm | ^8^ |
| $\boldsymbol{a}^{\boldsymbol{*}}$ | Mass specific area | 0.0008164 | dm^2^/mg | ^8^ |
| $\boldsymbol{k}_{\boldsymbol{d,}\boldsymbol{HID}}$ | Decay rate coefficient for $X_{\mathrm{HYD}}$ | 0.0006 | $d^{-1}$ | ^10^ |
| $\boldsymbol{k}_{\boldsymbol{d,}\boldsymbol{AUT}}$ | Decay rate coefficient for $X_{\mathrm{AUT}}$ | 0.0006 | $d^{-1}$ | ^10^ |
| $\boldsymbol{k}_{\boldsymbol{HID,STOB}}$ | Hydrolysis rate constant | 3 | $d^{-1}$ | ^11^ |
| $\boldsymbol{k}_{\boldsymbol{STOB,COD}}$ | Hydrolysis saturation constant | 1 | mg COD / mg COD | ^11^ |
| $\boldsymbol{K}_{\boldsymbol{HID,EPS}}$ | EPS hydrolysis rate coefficient | 0.1704 | $d^{-1}$ | ^4^ |
| $\boldsymbol{\eta}_{\boldsymbol{OX}}$ | Anoxic reduction factor | 0.6 |  | ^11^ |
| $\boldsymbol{\mu}_{\boldsymbol{HD,COD}}$ | Maximum growth rate of HD on COD | 5.76 | $d^{-1}$ | ^12^ |
| $\boldsymbol{\mu}_{\boldsymbol{HD,UAP}}$ | Maximum growth rate of HD on UAP | 1.272 | $d^{-1}$ | ^4^ |
| $\boldsymbol{\mu}_{\boldsymbol{HD,BAP}}$ | Maximum growth rate of HD on BAP | 0.0696 | $d^{-1}$ | ^4^ |
| $\boldsymbol{k}_{\boldsymbol{d,HD}}$ | Death rate coefficient of HD | 0.1992 | $d^{-1}$ | ^3^ |
| $\boldsymbol{k}_{\boldsymbol{N}\boldsymbol{O}_{\boldsymbol{2}}}^{\boldsymbol{HD}}$ | $S_{NO_{2}^{-}}$ affinity constant for HD | 0.5 | mg/l | ^3^ |
| $\boldsymbol{k}_{\boldsymbol{N}\boldsymbol{O}_{\boldsymbol{3}}}^{\boldsymbol{HD}}$ | $S_{NO_{3}^{-}}$ affinity constant for HD | 0.5 | mg/l | ^3^ |
| $\boldsymbol{k}_{\boldsymbol{COD,HD}}$ | biomass affinity constant for COD | 2 | mg/l | ^3^ |
| $\boldsymbol{k}_{\boldsymbol{UAP}}$ | biomass affinity constant for UAP | 100 | mg/l | ^4^ |
| $\boldsymbol{k}_{\boldsymbol{BAP}}$ | biomass affinity constant for BAP | 85 | mg/l | ^4^ |
| $\boldsymbol{\mu}_{\boldsymbol{SRB}}$ | Maximum growth rate of SRB | 0.55 | $d^{-1}$ | ^7^ |
| $\boldsymbol{k}_{\boldsymbol{COD}\boldsymbol{,}\boldsymbol{SRB}}$ | Half saturation value of SRB for COD | 6 | mg/l | ^7^ |
| $\boldsymbol{k}_{\boldsymbol{SRB}\boldsymbol{,}\boldsymbol{S}\boldsymbol{O}_{\boldsymbol{4}}}$ | Half saturation value of SRB for $S_{SO_{4}^{2-}}$ | 3.2 | mg/l | ^13^ |
| $\boldsymbol{k}_{\boldsymbol{d}\boldsymbol{,}\boldsymbol{SRB}}$ | Death rate coefficient of SRB | 0.02 | $d^{-1}$ | ^14^ |

# **References**

1. Ni, B. J., Ruscalleda, M. & Smets, B. F. Evaluation on the microbial interactions of anaerobic ammonium oxidizers and heterotrophs in Anammox biofilm. *Water Res.* **46**, 4645–4652 (2012).

2. Jiang, T. *et al.* Modelling the production and degradation of soluble microbial products (SMP) in membrane bioreactors (MBR). *Water Res.* **42**, 4955–4964 (2008).

3. Henze, M., Gujer, W., Mino, T. & van Loosedrecht, M. Activated Sludge Models ASM1, ASM2, ASM2d and ASM3. (2006) doi:10.2166/9781780402369.

4. Laspidou, C. S. & Rittmann, B. E. *Non-steady state modeling of extracellular polymeric substances, soluble microbial products, and active and inert biomass*. *Water Research* vol. 36 (2002).

5. Sierra-Alvarez, R. *et al.* Chemolithotrophic denitrification with elemental sulfur for groundwater treatment. *Water Res.* **41**, 1253–1262 (2007).

6. Xu, G., Yin, F., Chen, S., Xu, Y. & Yu, H. Q. Mathematical modeling of autotrophic denitrification (AD) process with sulphide as electron donor. *Water Res.* **91**, 225–234 (2016).

7. Kalyuzhnyi, S. V & Fedorovich, V. V. *MATHEMATICAL MODELLING OF COMPETITION BETWEEN SULPHATE REDUCTION AND METHANOGENESIS IN ANAEROBIC REACTORS*. *Bioresource Technology* vol. 65 (1998).

8. Kostrytsia, A. *et al.* Elemental sulfur-based autotrophic denitrification and denitritation: microbially catalyzed sulfur hydrolysis and nitrogen conversions. *J. Environ. Manage.* **211**, 313–322 (2018).

9. Liu, Y. *et al.* Evaluation of nitrous oxide emission from sulfide- and sulfur-based autotrophic denitrification processes. *Environ. Sci. Technol.* **50**, 9407–9415 (2016).

10. Sin, G. *et al.* Modelling nitrite in wastewater treatment systems: A discussion of different modelling concepts. *Water Sci. Technol.* **58**, 1155–1171 (2008).

11. Gujer, W., Henze, M., Mino, T. & Van Loosdrecht, M. Activated Sludge Model No. 3. in *Water Science and Technology* vol. 39 183–193 (No longer published by Elsevier, 1999).

12. Ni, B. J., Fang, F., Rittmann, B. E. & Yu, H. Q. Modeling microbial products in activated sludge under feast#famine conditions. *Environ. Sci. Technol.* **43**, 2489–2497 (2009).

13. Fedorovich, V., Lens, P. & Kalyuzhnyi, S. *Extension of Anaerobic Digestion Model No. 1 with Processes of Sulfate Reduction*. *Applied Biochemistry and Biotechnology* vol. 109 (2003).

14. Batstone, D. J. *et al.* The IWA Anaerobic Digestion Model No 1 (ADM1). *Water Sci. Technol.* **45**, 65–73 (2002).
